# Wider intraoperative glycemic fluctuation increases risk of acute kidney injury after pediatric cardiac surgery

**DOI:** 10.1080/0886022X.2018.1532908

**Published:** 2018-11-06

**Authors:** Guo-Huang Hu, Lian Duan, Meng Jiang, Cheng-Liang Zhang, Yan-Ying Duan

**Affiliations:** a Department of Surgery, The Fourth hospital of Changsha, Hunan Normal University, Changsha, China;; b Department of Cardiovascular Surgery, Xiangya Hospital, Central South University, Changsha, China;; c Department of Occupational and Environmental Health, Public Health School, Central South University, Changsha, China

**Keywords:** Acute kidney injury, intraoperative, glycemic fluctuation, cardiac surgery, pediatric

## Abstract

**Objective:** The association between poor intraoperative glycemic control and postoperative acute kidney injury (AKI) in adult cardiac surgery has been observed, but data in the pediatrics remain unknown. We performed a hypothesis that intraoperative hyperglycemia and/or wider glycemic fluctuation were associated with the incidence of postoperative AKI in pediatric cardiac surgery.

**Methods:** A retrospective study was performed in pediatrics who underwent cardiac surgery from 2013 to 2016. Perioperative glycemic data up to 48 hours after surgery were collected and analyzed. Patients with AKI were matched 1:1 with patients without AKI by a propensity score. Variables of demographic data, preoperative renal function and glycemic level, perioperative cardiac condition were matched.

**Results:** The incidence of AKI was 11.5% (118/1026), with 53.4% (63/118), 30.5% (36/118), and 16.1% (19/118) categorized as AKIN stages I, II, and III, respectively. Children who experienced AKI were younger and cyanotic, underwent more complex surgeries, had higher peak intraoperative glucose levels, wider intraoperative glycemic fluctuation, greater inotropic scores and more transfusions, and poor outcomes (all *p* < .05). After matching, the AKI group had significantly wider intraoperative glycemic fluctuation (*p* < .05). Logistic regression showed intraoperative glycemic fluctuation was one of the risk factors for AKI (*p* = .033) and degree of AKI severity stage increased when the glycemic fluctuation increased (*p* < .01).

**Conclusions:** Wider intraoperative glycemic fluctuation, but not hyperglycemia, was associated with an increased incidence of postoperative AKI after pediatric cardiac surgery.

## Introduction

Both hyperglycemia and glycemic fluctuation are frequently seen in the perioperative period and increase the risk of acute kidney injury (AKI) as described in the literature [1]. The mechanism of kidney functional damage is caused by exposure to transient hyperglycemia, most likely secondary to increased oxidative stress generated through the xanthine pathway observed in animal studies [2,3]. Acute hyperglycemia leads to increased urinary excretion of inflammatory cytokines/chemokines in humans with diabetes [4] or nondiabetes [5] and may directly contribute to kidney injury. Wider glycemic fluctuation is known to trigger inflammation to a higher degree than sustained hyperglycemia, leading to greater oxidative stress tissue damage [6,7].

The association between poor intraoperative glycemic control and postoperative AKI in cardiac surgery has been observed; intraoperative mean glucose >200 mg/dl was associated with increased renal morbidity [8], intraoperative mean glucose >180 mg/dl [9] or >150 mg/dl [10] was independently associated with AKI, maximal intraoperative glucose was associated with AKI [11] and glycemic variability was a predictor of AKI [9]. However, the results of interventional studies comparing tight or liberal blood glucose control in cardiac surgery conflict; tight blood glucose control (80–110 mg/dl) could reduce renal impairment [5,12] or not [11,13]. All of the abovementioned studies were adult samples and pediatric research has been very limited. Most congenital heart disease pediatric patients do not have diabetes or renal disease history, and lower age babies’ immature kidneys may make the results different from adults. Recently, Joshua’s [14] study showed that AKI pediatric patients after cardiac surgery had a large proportion of the highest intraoperative glucose >250 mg/dl. Furthermore, Andrew’s [15] study found that postoperative peak glucose in the cardiac intensive care unit (cICU) was associated with pediatric patients’ postoperative renal insufficiency, but the intraoperative peak glucose levels were not associated with postoperative renal insufficiency. However, the effect of intraoperative glycemic fluctuation was not addressed in the two studies. The association between how well intraoperative glycemic levels are controlled and postoperative renal function in children undergoing cardiac surgery is unclear. We performed a retrospective study to test the hypothesis that intraoperative hyperglycemia and/or wider glycemic fluctuation was associated with the incidence of postoperative AKI in pediatric cardiac surgery.

## Materials and methods

### Study population

After the study was approved by our hospital research committee, we reviewed hospital medical records, nursing records, the laboratory database, and the cardiac surgical database. Informed consent was waived because the study was a retrospective analysis. In total, 1026 pediatric patients (<18 years old) who underwent surgery for congenital heart disease from 1 January 2013, through 31 December 2016 were eligible for the analysis. Patients were excluded if they had: 1. preoperative kidney dysfunction or a history of renal/diabetic disease; 2. preoperative massive transfusion or nephrotoxic drug usage; 3. preoperative serious infection. Additionally, children were excluded if the daily medical record was incomplete, especially incomplete blood glycemic data less than 4 or incomplete sCr data less than 2 during the perioperative period. Furthermore, patients were excluded if their only operation did not involve cardiopulmonary bypass (CPB).

### Data acquisition

Researchers collected intraoperative glycemic data before, during, and after CPB and postoperative glycemic data up to 48 h after surgery. In this study, *initial glycemic level* (iG) is the first glycemic concentration measured during surgery but before CPB; *mean glycemic level* (mG) refers to the average of all perioperative glycemic levels for an individual; *peak glycemic level* (pG) is the highest glycemic level attained during surgery for each patient. The magnitude of *intraoperative glycemic fluctuation* (intraGF) was defined as the standard difference (SD) of each patient’s mean intraoperative glycemic level [16]. Patients were subdivided into three groups based on the magnitude of glycemic fluctuation: Group A: 0 to <36 mg/dl; Group B: ≥36 to 72 mg/dl; Group C: ≥72 mg/dl.

Pediatric AKI was defined according to a modified version of the Acute Kidney Injury Network (AKIN) criteria [17]. In brief, patients in this study were classified as having AKI if they had an increase in sCr to 1.5 times or more of their baseline within the first 48 h after the operation or to 26.5 μmol/L or more in the first 48 h after the operation. The severity stages are defined as follows: AKIN I, 1.5∼<2-fold baseline sCr；AKIN II, ≥2∼<3-fold baseline sCr；AKIN III, ≥3-fold baseline sCr. A patient’s baseline sCr was recorded during the hospitalization preceding the operation, and peak sCr was recorded within 48 h after the operation. We did not use the urine criteria in AKIN; urine output data that used retrospective ascertainment, including calculating the interval changes, would have a risk for inaccuracy.

All patients were cared by the same team of anesthesiologists, surgeons, perfusionists, and intensivists. Surgical complexity was assessed using the Risk Adjustment in Congenital Heart Surgery (RACHS)-1 score [18] because this was the most common case-mix adjustment method that was used when the trial protocol was developed. The details of anesthesia and the surgical procedure itself were reported previously [19]. Anesthesia was induced with intravenous midazolam and vecuronium bromide, followed by intravenous fentanyl and intermittent inhaled isoflurane to maintain anesthesia. A median sternal incision was made, and defects were repaired under CPB. Myocardial protection was achieved by intermittent perfusion of 4:1 cold blood cardioplegia or total cold crystalloid cardioplegia (custodial solution). CPB was performed using a pediatric hollow fiber oxygenator, circuits and a roller pump with nonpulsatile flow for both groups. Activated clotting time was maintained above 480 s, the perfusion flow was given at 80–150 mL/kg, the diluted hematocrit at 20–25% (neonates 30%) was gained by conventional priming program, and mild-to-moderate hypothermic (27–34 °C) was maintained during CPB. When necessary, hypo-perfusion flow (less than half of the targeted flow) or deep hypothermic circulation arrest (DHCA), multiple occlusion of the aorta (OA), and ultrafiltration were used during CPB period. After defect repairs were completed, rewarming started and ceased at a nasopharyngeal temperature of 37.0 °C and rectal temperature above 35.0 °C . Postoperatively, all patients were transported while they were intubated to the cICU. The postoperative inotropic score (IS _POD_) refers to all the inotropic drugs used from the heart rebeating time point to the postoperative 48 h and was calculated using the formula: (dopamine + dobutamine) × 1 +milrinone ×15 + (adrenaline + noradrenaline + isoproterenol) × 100. RBC _POD0-2_ refers to red blood cells (RBC) transfusion within postoperative 48 h, and Others _POD0-2_ refers to plasma(u)+platelet(u)+ cryoprecipitate(u) transfusion within postoperative 48 h. Fluid overload in the postoperative period was calculated by the formula: [total fluid administration (L) - total fluid output (L)]/basic weight(kg); results >5% were recorded [20].

Short-term outcomes that were evaluated included duration of postoperative mechanical ventilation (MV), length of cICU stay, hospital stay, renal replacement therapy (RRT: refer to postoperative peritoneal dialysis, blood dialysis or continuous blood purification) and mortality during hospitalization. 

### Statistical analyses

A Chi-square test was used for categorical variables that were summarized as frequencies and percentages. Continuous variables were expressed as the mean ± SD, and the *t*-test was used when data was normally distributed. The Mann–Whitney U-test was used when the data were non-normally distributed, and data were expressed as medians (IQR). To generate two evenly matched cohorts of patients who did and did not experience AKI, we propensity-scored children by using the following pre- and intra-operative variables: sex, age, weight, cyanosis, RACHS-1 score, preoperative left ventricular ejection fraction (LVEF), CPB time, OA time, iG, and baseline sCr. In the detection of 118 cases who had AKI, we matched 111 AKI patients with 111 control patients who underwent surgery during the same time period. The remaining seven cases had no suitable propensity scores. Variables associated with AKI with a *p* values <0.1 were then manually entered into a conditional logistic regression model to find the independent risk factors. The three groups of glycemic fluctuation and AKI severity stage were analyzed for linear trends. A *p* values <.05 was considered significant. Data analysis was performed using SPSS 23.0 statistical software (SPSS Inc., Chicago, IL).

## Results

During four years, the incidence of AKI was 11.5% (118/1026) in our pediatric cardiac surgery center. Of those with AKI, 53.4% (63/118), 30.5% (36/118), and 16.1% (19/118) were categorized as AKIN stages I, II, and III, respectively. The patients’ perioperative characteristics are shown in Table 1; diseases included but were not limited to tetralogy of Fallot with/without atrial septal defect, pulmonary atresia, anomalous pulmonary veins drainage, endocardial cushion defect, transposition of the great arteries, coronary artery fistula, aorta-pulmonary window, atrioventricular canal malformation, persistent truncus arteriosus, single ventricle or atrium, hypoplastic or interrupted aortic arch, and Ebstein or other valve anomalies. Before matching, the entire sample who experienced AKI were younger and cyanotic, had a higher RACHS-1 score, and less preoperative LVEF (all *p* < .01) when compared with those who did not experience AKI. They also underwent CPB for a longer time, longer OA time, more frequency of multiple OA, ultrafiltration, DHCA or hypoperfusion (all *p* < .01) during surgery. They had higher mG, pG, intraGF, IS _POD_, and more transfusions during the perioperative period (all *p* < .01).

**Table 1.** Patient perioperative characteristics and short-term outcomes with and without acute kidney injury in the entire sample and in the matched sample.

	Entire sample				Matched sample			
	No AKI (*n* = 908)	AKI (*n* = 118)	*χ*^2^（*Z* or *t*）	*p*	No CS-AKI (*n* = 111)	CS-AKI *(*n* =* 111)	*χ*^2^（*Z* or *t*）	*p*
Male sex [*n* (%)]	465 (51.2)	59 (50)	0.061	.804	53 (47.8)	58 (52.2)	0.649	.421
Age at surgery (mo), median (IQR)	36 (12,72)	12 (4,45)	(−5.726)	.000	12 (6,36)	13 (4,48)	(−0.139)	.889
Age ≤2 mo [*n* (%)]	37 (4.1)	15 (12.7)	16.191	.000	20 (18.0)	13 (11.7)	1.744	.187
Weight (kg), median (IQR)	13 (9,20)	8 (5,14.1)	(-6.169)	.000	8.5 (6.5,13)	8.5 (5.2,14.6)	(-0.440)	.660
Cyanotic [*n* (%)]	65 (7.2)	53 (44.9)	40.195	.000	50 (45.0)	47 (42.3)	0.165	.685
RACHS-1 score [*n* (%)]			136.667	.000			1.778	.777
1	91 (10.0)	1 (0.8)			1 (0.9)	1 (0.9)		
2	577 (63.5)	34 (28.9)			38 (34.2)	35 (31.5)		
3	190 (21.0)	44 (37.3)			44 (39.6)	43 (38.8)		
4	43 (4.7)	36 (30.5)			25 (22.6)	31 (27.9)		
5	7 (0.8)	3 (2.5)			3 (2.7)	1 (0.9)		
Preoperative LVEF <50% [*n* (%)]	225 (24.8)	52 (44.1)	19.712	.000	54 (48.6)	50 (45.0)	0.289	.591
Baseline sCr	43 (39,50)	41 (34,46)	(−1.083)	.279	42 (38,49)	41 (35,46)	(−0.974)	.330
CPB time (min), (x ± s )	72 ± 34	131 ± 83	(−7.665)	.000	110 ± 49	126 ± 82	(−1.718)	.087
OA time (min), (x ± s )	44 ± 26	77 ± 47	(−7.543)	.000	68 ± 29	75 ± 47	(−1.514)	.132
Multiple OA [*n* (%)]	14 (1.5)	15 (12.7)	47.438	.000	9 (8.1)	13 (11.7)	0.807	.369
Ultrafiltration [*n* (%)]	591 (65.1)	110 (93.2)	38.188	.000	102 (91.9)	100 (90.1)	0.220	.639
DHCA or hypoperfusion [*n* (%)]	17 (1.9)	15 (12.7)	40.608	.000	8 (7.2)	7 (6.3)	0.071	.789
iG (mg/dl), (x ± s )	86 ± 17	87 ± 20	(−0.965)	.335	92 ± 20	88 ± 20	(1.158)	.248
mG(mg/dl), (x ± s )	106 ± 25	124 ± 20	(−5.181)	.000	126 ± 30	126 ± 25	(0.170)	.865
pG(mg/dl), median (IQR)	134 (105,178)	163 (139,217)	(−5.758)	.000	162 (137,216)	162 (140,216)	(−0.695)	.487
IntraGF(mg/dl), median (IQR)	22 (13,38)	45 (25,76)	(−8.300)	.000	36 (20,63)	49 (25,81)	(−2.376)	.018
IS _POD_, median (IQR)	1(0,8)	7 (2,11)	(−6.269)	.000	2 (1,10)	7 (1,16)	(−2.961)	.003
Peak sCr	49 (42,56)	80 (64,110)	(−4.368)	.000	50 (43,55)	79 (65,112)	(−4.206)	.000
Transfusion: RBC _POD0-2_ (u), median (IQR)	0.5 (0,1.5)	1 (0,2.5)	(−5.234)	.000	0.5 (0,1.5)	1 (0,2)	(−2.540)	.011
Transfusion: Others _POD0-2_ (u), median (IQR)	0.5 (0,4)	2 (0,5)	(−4.102)	.000	2 (0,6)	2 (0,5)	(−0.647)	.518
FO_POD1_	105 (11.6)	16 (13.6)	0.400	.527	10 (9%)	15 (13.5%)	1.127	.288
FO_POD2_	91 (10.0)	12 (10.2)	0.002	.963	14 (12.6%)	11 (9.9%)	0.406	.524
Short-term outcomes								
Postoperative RRT [*n* (%)]	3 (0.3)	15 (12.7)	92.881	.000	0 (0)	13 (11.7)	13.809	.000
Length of MV(h), median (IQR)	14 (6,28)	36 (13,73)	(−5.925)	.000	15 (7,26)	34 (14,76)	(−4.257)	.000
Length of cICU stay(h), median (IQR)	70 (28,154)	119 (68,240)	(−5.145)	.000	68 (38,149)	157 (72,226)	(−4.857)	.000
Length of hospital stay(d), median (IQR)	12 (9,16)	15 (13,20)	(−6.921)	.000	12 (10,16)	16 (13,20)	(−4.991)	.000
Mortality [*n* (%)]	7 (0.8)	18 (15.3)	92.146	.000	0 (0)	15 (13.5)	16.087	.000

AKI: acute kidney injury; cICU: cardiac intensive care unit; CPB: cardiopulmonary bypass; DHCA: deep hypothermic circulation arrest; FO_POD1_: Fluid overload >5% within postoperative 24 h; FO_POD2_: Fluid overload >5% within postoperative 24–48 h; iG: initial glycemic level; intraGF: intraoperative glycemic fluctuation; IS_POD_: inotropic score to the postoperative 48 h; LVEF: left ventricular ejection fraction; MV: mechanical ventilation; mG: mean glycemic level; OA: occlusion of aorta; Others _POD0-2_: plasma(u)+platelet(u)+ cryoprecipitate(u) transfusion within postoperative 48 h; pG: peak glycemic level; RACHS-1: Risk Adjustment in Congenital Heart Surgery; RBC _POD0-2_: red blood cells transfusion within postoperative 48 h; RRT: renal replacement therapy; sCr: serum Creatinine.

The matched group set included 111 pairs of children with and without AKI. As illustrated in Table 1, the matched groups were balanced for the variables of demographic data, preoperative renal function, glycemic level, and preoperative and intraoperative cardiac condition. Compared with the entire sample, there Iwere no significant difference in pG or mG between the matched samples, but a significant difference was observed in intraGF, IS _POD_, and RBC _POD0-2_ transfusion between patients with AKI and those without AKI. Whether matched or not, the matched sample and the entire sample who had AKI had poor short-term outcomes including longer lengths of MV, cICU stay, hospital stay, as well as higher RRT and mortality (all *p* < .01).

**Figure 1.**
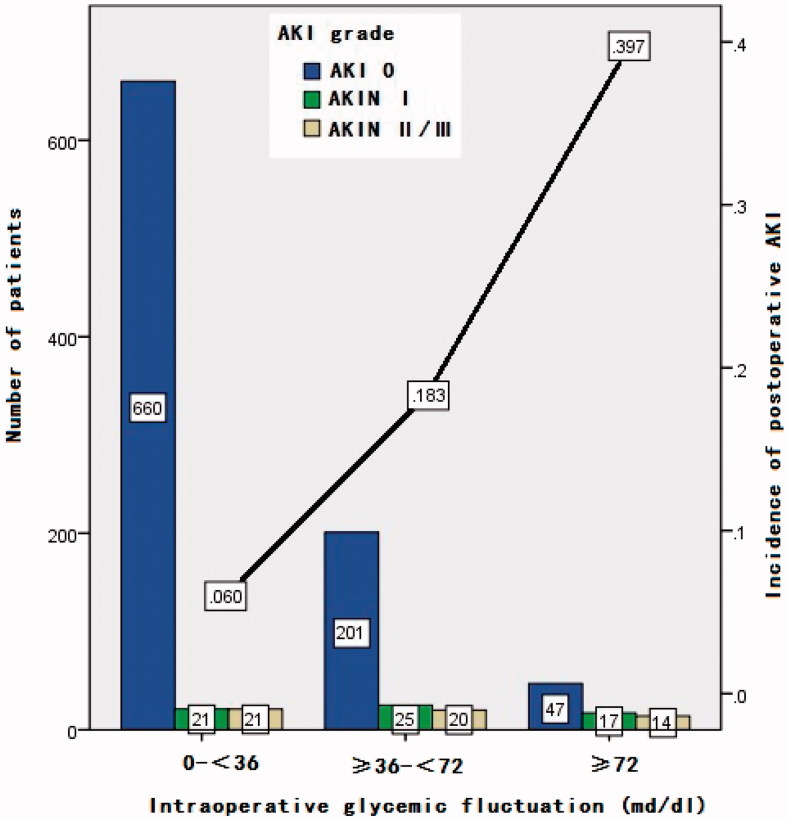
Intraoperative glycemic fluctuation and postoperatvie AKI (linear trend, *p* = .000). AKI: acute kidney injury; AKIN: acute kidney injury network.

The conditional logistic regression test showed intraGF (*p* = .033) and IS _POD_ (*p* = .031) were independent risk factors for AKI within the matched sample set (Table 2). The linear trend test (Figure 1) showed that the degree of AKI severity stage increased when the glycemic fluctuation increased; two were linear (Trend value 76.698, *p* = .000).

**Table 2.** Multivariate conditional logistic regression analysis of perioperative risk factors associated with AKI.

Variables	95%CI		HR	*p*
IntraGF	1.008	1.213	1.106	.033
IS _POD_	1.002	1.052	1.027	.031
Transfusion: RBC _POD0-2_	0.959	1.106	1.030	.417
CPB time	0.999	1.004	1.001	.224

CPB: cardiopulmonary bypass; intraGF: intraoperative glycemic fluctuation; IS_POD_: inotropic score to the postoperative 48 h; RBC_POD0-2_: red blood cells transfusion within postoperative 48 h.

## Discussion

Our study showed that wider intraGF was associated with postoperative AKI in pediatric cardiac CPB surgery. There was no association between perioperative mG or pG and postoperative AKI.

Our results demonstrated that the postoperative AKI incidence of cardiac surgery was 11.5%, consistent with a previous finding of 10–45% based on AKIN criteria [16,21]. The 1.8% rate of requiring RRT is consistent with the 1–5% that was previously reported [22]. Potential preoperative and intraoperative factors contributing to the difference in AKI frequency [23], such as younger age, small body size, higher RACHS-1 score and CPB strategy were matched 1:1 by the propensity score between patients who had AKI and those who did not. We found that patients with AKI still had higher intraGF after propensity matching the above variables.

The current consensus regarding glycemic control in the critically ill is that strict glycemic control is not beneficial and may even be harmful [24]. Glycemic fluctuation in critically ill patients has recently gained interest and has repeatedly been shown to be associated with adverse outcomes [25]. It is increasingly recognized that acute glycemic variability is a more specific trigger of oxidative stress, when compared with chronic sustained hyperglycemia states. Glycemic fluctuation increases AKI risk in adult patients undergoing coronary artery bypass graft surgery [26], but data in pediatric patients remain unknown. However, our study suggested that children who underwent cardiac surgery with more intraoperative glycemic fluctuation were more likely to develop postoperative AKI compared with patients who had less intraoperative glycemic fluctuation. Furthermore, the degree of AKI severity stage increased when glycemic fluctuation increased. The association between glycemic fluctuation and AKI can be explained by a plausible biological explanation [6,7] that intermittent hyperglycemia causes more oxidative stress in endothelial cells compared to constant hyperglycemia and induces a greater over-production of reactive oxygen species by NADPH. Krinsley’s study [27] showed that nondiabetics were less tolerant to acute glycemic fluctuation because of their lack of chronic exposure to these variabilities. Bansal’s study [9] showed that glycemic fluctuation was a predictor of an increase in creatinine and AKI after cardiac surgery. Although there is currently insufficient evidence to ascertain the mechanism of this phenomenon, our data strengthened this finding.

There are some negative findings in our study. Fluid overload is known [27,28] as a risk factor that is associated with AKI incidence and degree, hospital duration and mortality. Even mild fluid overload is an independent risk factor for the incidence of AKI [29]. Our results did not find a significant difference between fluid overload (>5%) and AKI incidence within 48-h postoperation (Table 1), most likely because most of our patients underwent intraoperative ultrafiltration (entire sample >65% while matched sample >90%) and ultrafiltration-related volume depletion modified postoperative fluid overload to a certain extent. DHCA or hypoperfusion in CPB [30,31] is another risk factor of postoperative AKI in some animal and human studies, especially in adult aortic surgeries [32,33]. However, our results showed no significant difference of DHCA and AKI incidence; a plausible explanation is that DHCA incidence (6–7% in matched pairs) is not large enough to have a significant difference and other CPB strategies (such as hypothermia, sufficient oxygen supply, slowly and equably rewarming) protect kidney function.

Our study has a number of limitations. First, it is a retrospective single-center observational study, and information bias should be considered. A prospective study is warranted to confirm our results. Second, intermittent measurements of glycemia and sCr 48 h perioperatively may have underestimated hyperglycemia or AKI incidence. Third, drugs that affect renal function or glycemic level (such as antibiotics, diuretics and insulin) that were administered were not included in the analysis. Fourth, the study comprised patients of different ages with heterogeneous congenital heart defects; this increases the heterogeneity but improves the generalizability of the study. We balanced the heterogeneity of patient and operation characteristics with propensity score matching.

## Conclusions

In our retrospective single-center observational study, wider intraoperative glycemic fluctuation, but not hyperglycemia, seems to be associated with an increased incidence of postoperative AKI after pediatric cardiac surgery. Further prospective trials are mandated to confirm the association found in our study.

## Ethical approval

This article does not contain any studies with human participants or animals performed by any of the authors. The study was approved by our hospital research committee. We reviewed hospital medical records, nursing records, the laboratory database, and the cardiac surgical database. Informed consent was waived because the study was a retrospective analysis.

## References

[1] Mendez CE, Der Mesropian PJ, Mathew RO, et al. Hyperglycemia and acute kidney injury during the perioperative period. Curr Diab Rep. 2016;16:10. 26792142 10.1007/s11892-015-0701-7

[2] Vanhorebeek I, Gunst J, Ellger B, et al. Hyperglycemic kidney damage in an animal model of prolonged critical illness. Kidney Int. 2009;76:512–520. 19536085 10.1038/ki.2009.217

[3] Ryutaro H, Xu F, Dang K, et al. Transient hyperglycemia affects the extent of ischemia-reperfusion-induced renal injury in rats. Anesthesiology. 2008;108:402–414. 18292678 10.1097/ALN.0b013e318164cff8

[4] Cherney DZJ, Scholey JW, Sochett E, et al. The acute effect of clamped hyperglycemia on the urinary excretion of inflammatory cytokines/chemokines in uncomplicated type 1 diabetes: a pilot Study. Diab Care. 2011;34:177–180. 10.2337/dc10-1219 PMC3005484 20841614

[5] Patrick L, Van Vlem B, Coddens J, et al. Tight perioperative glucose control is associated with a reduction in renal impairment and renal failure in non-diabetic cardiac surgical patients. Crit Care. 2008;12:R154. 19055829 10.1186/cc7145 PMC2646319

[6] Ge QM, Dong Y, Zhang HM, et al. Effects of Intermittent high glucose on oxidative stress in endothelial cells. Acta Diabetol. 2010;47:97–103. 10.1007/s00592-009-0140-5 19763390

[7] Morihiko M, Hayashi T, Mizuno N, et al. Intermittent high glucose implements stress-induced senescence in human vascular endothelial cells: role of superoxide production by NADPH oxidase. PLoS One. 2015;10:e0123169. 25879533 10.1371/journal.pone.0123169 PMC4400006

[8] Andra E, Abd-Elsayed A, Maheshwari A, et al. Role of intraoperative and postoperative blood glucose concentrations in predicting outcomes after cardiac surgery. Anesthesiology. 2010;112:860–871. 20216389 10.1097/ALN.0b013e3181d3d4b4

[9] Bansal B, Carvalho P, Mehta Y, et al. Prognostic significance of glycemic variability after cardiac surgery. J Diab Comp. 2016;30:613–617. 10.1016/j.jdiacomp.2016.02.010 26965795

[10] Song JW, Shim JK, Yoo KJ, et al. Impact of intraoperative hyperglycaemia on renal dysfunction after off-pump coronary artery bypass. Interact Cardiovasc Thorac Surg. 2013;17:473–478. 23690431 10.1093/icvts/ivt209 PMC3745134

[11] Gunjan YG, Nuttall GA, Abel M, et al. Intraoperative hyperglycemia and perioperative outcomes in cardiac surgery patients. Mayo Clin Proc. 2005;80:862–866. 16007890 10.4065/80.7.862

[12] van den Berghe G, Wouters P, Weekers F, et al. Intensive insulin therapy in critically ill patients. N Engl J Med. 2001;345:1359–1367. 11794168 10.1056/NEJMoa011300

[13] Raquel P, Galas F, Hajjar L, et al. Intensive perioperative glucose control does not improve outcomes of patients submitted to open-heart surgery: a randomized controlled trial. Clinics. 2009;64:51–60. 19142552 10.1590/S1807-59322009000100010 PMC2671976

[14] Joshua JB, Asaro LA, Wypij D, et al. Acute kidney injury after pediatric cardiac surgery: a secondary analysis of the safe pediatric euglycemia after cardiac surgery trial. Pediatr Crit Care. 2017;18:638–646. 10.1097/PCC.0000000000001185 PMC5503840 28492399

[15] Andrew RY, Dyke PC, Taeed R, et al. Hyperglycemia is a marker for poor outcome in the postoperative pediatric cardiac patient. Pediatr Crit Care. 2006;7:351–355. 10.1097/01.PCC.0000227755.96700.98 16738506

[16] Christian W, Schnell O The assessment of glycemic variability and its impact on diabetes-related complications: an overview. Diabetes Technol. 2009;11:623–633. 10.1089/dia.2009.0043 19821754

[17] Duan L, Hu GH, Jiang M, et al. Clinical characteristics and prognostic analysis of children with congenital heart disease complicated by postoperative acute kidney injury. Chin J Comtemp Pediatr. 2017;19:1196–1201. 10.7499/j.issn.1008-8830.2017.11.014 PMC7389321 29132469

[18] Kathy JJ, Koch KJ, Owens PL, et al. Development and validation of an agency for healthcare research and quality indicator for mortality after congenital heart surgery harmonized with risk adjustment for congenital heart surgery (RACHS-1) methodology. J Am Heart Assoc. 2016;5:e003028. 27207997 10.1161/JAHA.115.003028 PMC4889177

[19] Luo WJ, Zhu M, Huang RM, et al. A comparison of cardiac post-conditioning and remote pre-conditioning in paediatric cardiac surgery. Cardiol Young. 2011;21:266–270. 21262079 10.1017/S1047951110001915

[20] David M, Goldstein S, Cooper D, et al. Peritoneal dialysis vs furosemide for prevention of fluid overload in infants after cardiac surgery: a randomized clinical trial. JAMA Pediatr. 2017;171:357–364. 28241247 10.1001/jamapediatrics.2016.4538

[21] Tanyildiz M, Ekim M, Kendirli T, et al. Acute kidney injury in congenital cardiac surgery: pediatric risk-injury-failure-loss-end-stage renal disease and Acute Kidney Injury Network. Pediatr Int. 2017; 59:1252–1260. 28672079 10.1111/ped.13359

[22] Conlon PJ, Stafford-Smith M, White WD, et al. Acute renal failure following cardiac surgery. Nephrol Dial Transplant. 1999;14:1158–1162. 10344355 10.1093/ndt/14.5.1158

[23] Daishi H, Ito A, Yamada A, et al. Independent risk factors and 2-year outcomes of acute kidney injury after surgery for congenital heart disease. Am J Nephrol. 2017;46:204–209. 28858859 10.1159/000480358

[24] The NICE-SUGAR Study Investigators Hypoglycemia and risk of death in critically ill patients. N Engl J Med. 2012; 367:1108–1118. 22992074 10.1056/NEJMoa1204942

[25] Krinsley JS Glycemic variability: a strong independent predictor of mortality in critically ill patients. Crit Care Med. 2008;36:3008–3013. 18824908 10.1097/CCM.0b013e31818b38d2

[26] Ming AS, Liu W, Ng R, et al. Wider perioperative glycemic fluctuations increase risk of postoperative acute kidney injury: a prospective cohort study. Med. 2015;94:e1953. 10.1097/MD.0000000000001953 PMC4915904 26554803

[27] John RP, Christopher JK, et al. Fluid management for the prevention and attenuation of acute kidney injury. Nat Rev Nephro. 2014;10:37–47. 10.1038/nrneph.2013.232 24217464

[28] Scott MS, Zappitelli M, Alexander S, et al. Fluid overload and mortality in children receiving continuous renal replacement therapy: the prospective pediatric continuous renal replacement therapy registry. Am J Kidney Dis. 2010;55:316–325. 20042260 10.1053/j.ajkd.2009.10.048

[29] The Beijing Acute Kidney Injury Trial (BAKIT) Workgroup Fluid balance and mortality in critically ill patients with acute kidney injury: a multicenter prospective epidemiological study. Crit Care. 2015;19:371. 26494153 10.1186/s13054-015-1085-4 PMC4619072

[30] Maite AG, Alcaraz RA, Romero OA, et al. Prognostic relevance of early AKI according to PRIFLE criteria in children undergoing cardiac surgery. Pediatr Nephrol. 2014;29:1265–1272. 24496588 10.1007/s00467-014-2757-z

[31] Lei Y, Gu T, Zhang G, et al. The deep hypothermic circulatory arrest causes more kidney malfunctions based on a novel rabbit model. Ann Saudi Med. 2014;34:532–540. 25971829 10.5144/0256-4947.2014.532 PMC6074567

[32] Kim WH, Park MH, Kim HJ, et al. Potentially modifiable risk factors for acute kidney injury after surgery on the thoracic aorta: a propensity score matched case-control study. Med. 2015;94(2):e273. 10.1097/MD.0000000000000273 PMC4602544 25590836

[33] Mori Y, Sato N, Kobayashi Y, et al. Acute kidney injury during aortic arch surgery under deep hypothermic circulatory arrest. J Anesth. 2011;25:799–804. 21847704 10.1007/s00540-011-1210-8

